# Cross-neutralization and cross-protection among SARS-CoV-2 viruses bearing different variant spikes

**DOI:** 10.1038/s41392-022-01137-1

**Published:** 2022-08-13

**Authors:** Yang Liu, Jianying Liu, Jing Zou, Birte Kalveram, Rafael R. G. Machado, Ping Ren, Sina Türeli, Derek J. Smith, Scott C. Weaver, Xuping Xie, Pei-Yong Shi

**Affiliations:** 1 Department of Biochemistry and Molecular Biology, University of Texas Medical Branch, Galveston, TX USA; 2 Institute of Infectious Diseases, Shenzhen Bay Laboratory, Shenzhen, China; 3 Department of Microbiology and Immunology, University of Texas Medical Branch, Galveston, TX USA; 4 Department of Pathology, University of Texas Medical Branch, Galveston, TX USA; 5 Center for Pathogen Evolution, Department of Zoology, University of Cambridge, Cambridge, UK; 6 Sealy Institute for Drug Discovery, University of Texas Medical Branch, Galveston, TX, USA; 7 Sealy Center for Structural Biology & Molecular Biophysics, University of Texas Medical Branch, Galveston, TX, USA

**Keywords:** Vaccines, Microbiology

**Dear Editor**

The rapid evolution of SARS-CoV-2 mandates a better understanding of cross-neutralization and cross-protection among variants. Such information is essential to guide vaccine strategy and public policy. To examine the cross-protection among different variant spikes, we initially prepared four chimeric SARS-CoV-2 viruses (Fig. 1a), each bearing the spike gene from Alpha, Beta, Gamma, or Epsilon in the backbone of USA-WA1/2020 [isolated in January 2020 and defined as wild-type (WT)]. The four variants were selected based on their high prevalence at the onset of the project, when Delta and Omicron had not emerged. Each variant spike contained a distinct set of mutations (Fig. 1a). An additional substitution E484K was added to the original Alpha variant (Alpha + E484K-spike) as this mutation occurred in many clinical isolates.^1^ The spike genes from all recombinant viruses were sequenced to ensure no aberrant mutations. Comparable ratios of viral RNA copies versus plaque-forming units (RNA/PFU) were obtained for both WT and chimeric viruses when produced and analyzed on Vero E6 cells (Supplementary Fig. 1), suggesting equivalent specific infectivity of the viral stocks.

**Fig. 1:**
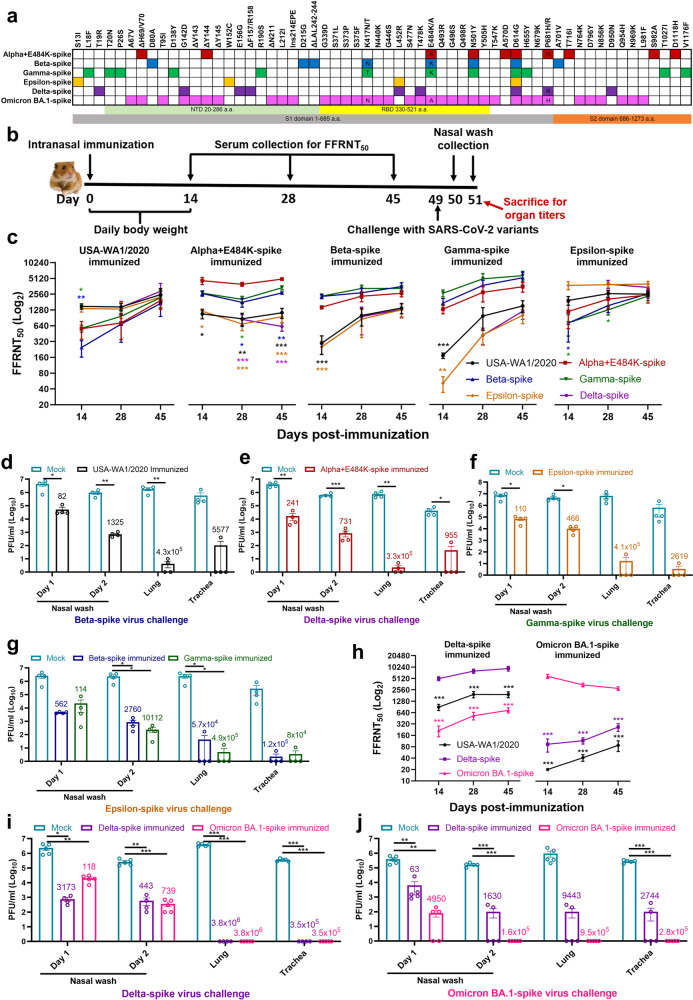
Cross-neutralization and corss-protection among different SARS-CoV-2 variants in hamsters. **a** Amino acid substitutions in variant spikes. The spike sequence from USA-WA1/2020 strain was used as a reference. NTD N-terminal domain, RBD receptor-binding domain. **b** Experimental scheme. The hamsters (*n* = 4 or 5 per group) were intranasally immunized with WT or variant-spike SARS-CoV-2 (10^6^ PFU). Serum NT_50_ values were measured on days 14, 28, and 45 post-infection. On day 49 post-infection, the hamsters were intranasally challenged by indicated variant-spike SARS-CoV-2 (10^4^ PFU). The nasal washes (NW) were quantified for viral titers on days 1 and 2 post-challenge. Viral loads in lungs and trachaes were measured on day 2 post-challenge. **c–g** Cross-neutralization and corss-protection among Alpha, Beta, Gamma, Epsilon, or Delta. **h–j** Cross-neutralization and corss-protection between Delta and Omicron (BA.1). **c**, **h** Neutralizing titers of sera against variant spikes on days 14, 28, and 45 post-immunization. The values in the graph represent the mean ± standard error of mean. An unpaired two-tailed *t* test was used to determine significant differences between self-neutralization and cross-neutralization groups. *P* values were adjusted using the Bonferroni correction to account for multiple comparisons. Differences were considered significant if *p* < 0.01; *p* < 0.01, *; *p* < 0.002, **; and *p* < 0.0002, *** (**c**) or *p* < 0.025; *p* < 0.025, *; *p* < 0.005, **; and *p* < 0.0005, *** (**h**). **d–g** and **i–j** Cross-protection against heterologous spike variants. The non-immunized mock and immunized hamsters were challenged with selected variant spike viruses. The viral loads in nasal wash, lung, and trachea were quantified by plaque assays. The numbers above individual columns indicate the fold decrease in viral loads by comparing the means from the immunized group with that from the non-immunized group. Means ± standard errors of the mean are shown. An unpaired two-tailed *t* test was used to determine significant differences between mock and immunized groups. Differences were considered significant if *p* < 0.05; *p* < 0.05, *; *p* < 0.01, **; and *p* < 0.001, *** . **d–f** *P* values were adjusted using the Bonferroni correction to account for multiple comparisons. Differences were considered significant if *p* < 0.025; *p* < 0.025, *; *p* < 0.005, **; and *p* < 0.0005, *** (**g, i–j**)

To analyze the immunogenicity of different variant spikes, we intranasally infected hamsters with 10^6^ PFU of recombinant WT or variant-spike virus (Fig. 1b). The infected animals developed different degrees of weight loss in the order of Alpha+E484K-spike > Beta-spike ≈ Gamma-spike > WT ≈ Epsilon-spike (Supplementary Fig 2a). The weight loss results were consistent with the disease scores, with the Alpha+E484K-spike virus causing the most severe disease (Supplementary Fig 2b). These results suggest that Alpha+E484K-spike is the most pathogenic virus in hamsters. Sera were collected on days 14, 28, and 45 post-immunization and measured for neutralizing titers against homologous and heterologous variant-spike viruses, including the Delta-spike virus that emerged when the experiment was performed (Fig. 1b). To increase assay throughput, we developed a “fluorescent foci” reduction neutralization test (FFRNT) by using mNeonGreen (mNG) chimeric-spike viruses (Supplementary Fig 3a). The mNG gene was engineered into the open-reading-frame-7 (ORF7) of the viral genome.^2^ The protocols for the conventional plaque reduction neutralization test (PRNT) and FFRNT (supplementary Fig 3b) were similar except that the latter quantifies “fluorescent Foci” using a high-content imager in a high-throughput manner (supplementary Fig 3c). The two assays yielded comparable neutralizing titers for the same set of BNT162b2-vaccinated human sera (Fig. S3d–e), validating the utility of FFRNT for neutralization test.

FFRNT analysis of immunized hamster sera showed distinct neutralizing profiles against homologous and heterologous SARS-CoV-2 variants (Summary in Fig. 1c and details in supplementary Fig. 4 and supplementary Tables 1–3). Each variant spike elicited faster and higher neutralizing titers against its homologous SARS-CoV-2 variant than heterologous variants. The magnitudes and ranks of neutralizing titers against different heterologous variants varied depending on the infected variant spikes. Unlike other variant spike-immunized groups, the Alpha-spike-infected animals did not seem to increase the neutralizing titers against heterologous variants from days 14 to 45 post-infection. Notably, from days 14 to 45 post-infection, homologous neutralization titers increased by ≤2.32-fold, whereas heterologous neutralization titers could increase up to 22-fold when Gamma-spike-infected sera were tested against epsilon-spike SARS-CoV-2 (Fig. 1c). On days 14, 28, and 45 post-infection, the differences in neutralizing titers between homologous and heterologous variants could be as large as 62-, 15-, 9.7-fold, respectively (Fig. 1c). Collectively, the results demonstrate that infection of hamsters with USA-WA1/2020 bearing different variant spikes elicits distinct kinetics, magnitudes, and ranks of neutralizing titers against homologous and heterologous variants.

Using the neutralization data in supplementary Tables 1–3, we created three antigenic maps to visualize the relationships between sera collected from infected hamsters at days 14, 28, and 45 against SARS-CoV-2 variants (Supplementary Fig 5). FFRNT titers were used to position the serum relative to each virus using antigenic cartography (a modification of multidimensional scaling for binding assay data) such that higher neutralization titers were represented by shorter distances between serum and the virus.^3^ Each gridline or antigenic unit of the map corresponds to a twofold difference in neutralization titer of a given virus. In all cases, two clusters were observed for both antigens and sera: one containing WT USA-WA1/2020, Delta, and Epsilon; the other Alpha+E484K, Beta, and Gamma. Comparison of maps for Days 14 and 45 also revealed the increasing cross-reactivity of WT USA-WA1/2020 and Epsilon sera as distances between clusters decrease and WT USA-WA1/2020 and Epsilon sera became more equidistant to these two clusters.

To evaluate cross-protection, we selected variant viruses exhibiting the lowest neutralizing titers for each immunized group to challenge the hamsters on day 49 post-infection. Specifically, animals immunized with WT, Alpha-, Epsilon-, Beta-, or Gamma-spike were challenged with 10^4^ PFU of Beta-, Delta-, Gamma-, Epsilon-, or Epsilon-spike SARS-CoV-2 viruses, respectively. Compared with PBS-immunized, challenged animals, all variant spike-immunized hamsters were protected from the challenge and developed significantly lower viral loads in nasal washes (82- to 10,112-fold), tracheas (955- to 120,000-fold), and lungs (57,000- to 490,000-fold) (Fig. 1d–g).

After the completion of the above experiments, Omicron varint emerged. Since Omicron exhibited the least sensitivity to vaccine-elicited neutralization among all known variants^4^ and became globally prevalent, we examined the cross-neutralization and cross-protection between these two variants. A chimeric USA/WA1-2020 bearing the complete spike from Omicron BA.1 was constructed, resulting in Omicron BA.1-spike SARS-CoV-2 (Fig. 1a). Hamsters were intranasally inoculated with 10^6^ PFU of Delta- or Omicron BA.1-spike virus. The Delta-spike virus-innoculted animals developed more weight loss (Supplementary Fig 6a) and diseases (Supplementary Fig 6b) than the Omicron BA.1-spike virus-inoculated hamsters. The results are in agreement with the recent reports that Omicron causes milder disease in hamsters^5^ and humans.^6^

For cross-neutralization, Delta-spike virus elicited neutralization titers in the order of Delta-spike > WT USA/WA1/2020 > Omicron BA.1-spike virus; on day 28 post-immunization, the neutralization titer against Delta-spike virus was 4.1- and 15.1-fold higher than those of WT and Omicron BA.1-spike virus, respectively (Fig. 1h, left panel, Supplementary Fig 7a and Supplementary Table 4). Although the Delta-spike virus-immunized animals were protected against challenges of both Delta- and Omicron BA.1-spike viruses, the protection against Delta-spike virus was stronger than that against Omicron BA.1-spike virus (Fig. 1i–j).

The Omicron BA.1-spike virus elicited neutralization titers in the order of Omicron BA.1-spike > Delta-spike > WT virus; remarkably, on day 28 post-immunization, the neutralization titer against Omicron BA.1-spike virus was 40.1- and 127.2-fold higher than those of Delta-spike and WT virus, respectively (Fig. 1h right panel, supplementary Fig 7b and supplementary Table 4). The neutralization differences between the homologous and heterologous viruses are much larger from the Omicron BA.1-spike virus-infected group than those from the Delta-spike virus-infected group (Fig. 1h). Infection with Omicron BA.1-spike virus protected hamsters against both Delta- and Omicron BA.1-spike viruses (Fig. i–j). The results again demonstrate that different variant spikes elicit distinct cross-neutralization profiles after SARS-CoV-2 infection, leading to different levels of cross-protection against other variants.^7^

The heterogeneity of cross-neutralization among different variants may have implications in vaccine strategy. However, when applying our results to vaccine strategy, this study has two limitations. First, we used chimeric viruses rather than clinically approved vaccine platforms (e.g., mRNA) for expressing variant spikes. The approved vaccine platforms with variant spikes were not available for research labs for head-to-head comparison studies. The neutralizing profile elicited by chimeric viruses could differ from that elicited by the clinically approved vaccine platforms. In chimeric virus-infected hamsters, immune responses to non-spike viral proteins may provide additional protection when compared with animals immunized with spike-alone vaccines. Second, we did not analyze T cell and other types of immunity, which are known important for protection. Unlike neutraliztion that could be significantly evaded by variants, the majority of T cell epitopes are highly preserved against varinat spikes (including Omicron) after vaccination or infection.^8^ Despite these limitations, our study has systematically uncovered the cross-neutralization and cross-protection among different variants in a hamster model; many of these variant infection-and-challenge combinations are impossible to achieve in COVID-19 patients (e.g., Omicron infection followed by Delta challenge).

## Supplementary information

Supplementary information

## Data Availability

All data reported in this paper will be shared by the lead contact upon request. This paper does not report original code. Any other information required to reanalyze the data reported in this paper are available upon request.
