# Supplementary material for: Cross-neutralization and cross-protection among SARS-CoV-2 viruses bearing different variant spikes
Source: Signal Transduct Target Ther. 2022 Aug 13;7:285. doi: 10.1038/s41392-022-01137-1 (PMC9375067; doi:10.1038/s41392-022-01137-1)
Supplement: Supplementary file 1 — Supplementary information [file 41392_2022_1137_MOESM1_ESM.docx]

**Cross-neutralization and cross-protection among SARS-CoV-2 viruses bearing different variant spikes**

Yang Liu^1,7*^, Jianying Liu^2,7*^, Jing Zou^1,*^, [Birte Kalveram](https://pubmed.ncbi.nlm.nih.gov/?sort=date&term=Kalveram+B&cauthor_id=34516560)^1,2^, Rafael R. G. Machado^2^, Ping Ren^3^, Sina Türeli^4^, Derek J. Smith^4^, Scott C.Weaver^2^, Xuping Xie^1,#^, Pei-Yong Shi^1,5,6,#^

^1^Department of Biochemistry and Molecular Biology, University of Texas Medical Branch, Galveston TX, U.S.A.

^2^Department of Microbiology and Immunology, University of Texas Medical Branch, Galveston TX, U.S.A.

^3^Department of Pathology, University of Texas Medical Branch, Galveston, TX, U.S.A.

^4^Center for Pathogen Evolution, Department of Zoology, University of Cambridge, Cambridge, UK

^5^Sealy Institute for Drug Discovery, University of Texas Medical Branch, Galveston, TX, USA

^6^Sealy Center for Structural Biology & Molecular Biophysics, University of Texas Medical Branch, Galveston, TX, USA

^7^Institute of Infectious Diseases, Shenzhen Bay Laboratory, Shenzhen, China

^*^ These authors contributed equally to this work.

^#^Correspondence: X.X. (xuxie@UTMB.edu) or P.-Y.S. ([peshi@UTMB.edu](mailto:peshi@UTMB.edu))

**This PDF file includes:**

Materials and Methods

Supplementary figures 1 to 7

Supplementary tables 1 to 4

**Materials and Methods**

**Ethics statement.** Hamster studies were performed under the guidance of the Care and Use of Laboratory Animals of the University of Texas Medical Branch (UTMB). The protocol (2009087) was approved by the Institutional Animal Care and Use Committee (IACUC) at UTMB. All the hamster operations were performed under anesthesia by isoflurane to minimize animal suffering.

**Animals and Cells.** The Syrian golden hamsters (HsdHan:AURA strain) were purchased from Envigo (Indianapolis, IN). Vero E6 cells, an African green monkey kidney epithelial cell line (ATCC, Manassas, VA, USA), were cultured in Dulbecco’s modified Eagle’s medium (DMEM; Gibco/Thermo Fisher, Waltham, MA, USA) with 10% fetal bovine serum (FBS; HyClone Laboratories, South Logan, UT) plus 1% ampicillin/streptomycin (Gibco). The authenticity of Vero E6 cells was verified using Short Tandem Repeat profiling by ATCC. The cells were tested negative for mycoplasma.

**Construction of chimeric SARS-CoV-2 viruses with variant spikes and mNeonGreen (mNG) reporter viruses.** All spike mutations from different variants were engineered into an infectious cDNA clone of an early SARS-CoV-2 isolate USA-WA1/2020 (MT020880) using a standard PCR-based mutagenesis method. The protocol for the construction of recombinant SARS-CoV-2 was reported previously^1,2^. To construct the mNG reporter viruses with variant spikes, the mNG gene was engineered into the open-reading-frame-7 (ORF7) of the viral genome. The full-length cDNAs of the viral genome containing the variant spike mutations were assembled by *in vitro* ligation. The resulting genome-length cDNAs served as templates for *in vitro* transcription of full-length viral RNAs. The full-length viral RNA transcripts were electroporated into Vero E6 cells. On day 2 post electroporation (when the electroporated cells developed cytopathic effects due to recombinant virus production and replication), the original viral stocks (P0) were harvested from the culture medium. The P0 viruses were amplified on Vero E6 cells for another round to produce working viral stocks (P1). The complete spike genes from the P1 viruses were sequenced to ensure no undesired mutations. The P1 viruses were used for the following studies. The construction and characterization of Omicron BA.1-spike SARS-CoV-2 was recently reported^3,4^. Omicron spike from sublineage BA.1 was used in the study.

**Plaque assay.** Approximately 1.2×10^6^ Vero E6 cells were seeded to each well of 6-well plates and cultured at 37°C, 5% CO_2_ for 16 h. The virus was serially diluted in DMEM with 2% FBS and 200 µl diluted viruses were transferred onto the monolayers. The viruses were incubated with the cells at 37°C with 5% CO_2_ for 1 h. After the incubation, 2 ml of overlay medium was added to the infected cells per well. The overlay medium contained DMEM with 2% FBS, 1% penicillin/streptomycin, and 1% sea-plaque agarose (Lonza, Walkersville, MD). After a 2-day incubation, plates were stained with neutral red (Sigma-Aldrich, St. Louis, MO) and plaques were counted on a lightbox.

**Quantitative real-time RT-PCR assays.** RNA copies of SARS-CoV-2 samples were detected by quantitative real-time RT-PCR (RT-qPCR) assays using the iTaq SYBR Green One-Step Kit (Bio-Rad) on the LightCycler 480 system (Roche, Indianapolis, IN) following the manufacturer's protocols. The absolute quantification of viral RNA was determined by a standard curve method using an RNA standard (*in vitro* transcribed 3,840 bp containing genomic nucleotide positions 26,044 to 29,883 of SARS-CoV-2 genome).

**Hamster infections.** Four- to six-week-old male golden Syrian hamsters, strain HsdHan:AURA (Envigo, Indianapolis, IN), were intranasally immunized with 10^6^ PFU recombinant WT or variant spike virus on day 0. The immunized animals were weighed and monitored for signs of illness daily. Sera were collected on days 14, 28, and 45 post-immunization and measured for neutralizing titers against homologous and heterologous variant-spike viruses. On day 49, animals from each immunized group were challenged with 10^4^ PFU of selected variant viruses exhibiting the lowest neutralizing titers, as indicated in **Figure 1**. Specifically, animals immunized with WT, Alpha-, Beta-, Gamma-, Epsilon-, Delta-, or Omicron BA.1-spike were challenged with the Beta-, Delta-, Epsilon-, Epsilon-, Gamma-, Delta-, or Omicron BA.1-spike SARS-CoV-2, respectively. Nasal washes were collected in 400 µl sterile DPBS at indicated time points. Animals were humanely euthanized for organ collections after 2 days of the challenge. The harvested tracheae and lungs were placed in a 2-ml homogenizer tube containing 1 ml of maintenance media (DMEM supplemented with 2% FBS and 1% penicillin/streptomycin) and stored at -80°C. Samples were subsequently thawed, lung or tracheae were homogenized using TissueLyser II (Qiagen, Hilden, Germany) for 1 min at 26 sec-1, and debris was pelleted by centrifugation for 5 min at 16,100×g. Infectious titers were determined by plaque assay.

**Human serum specimens.** The research protocol regarding the use of human serum specimens was reviewed and approved by the University of Texas Medical Branch (UTMB) Institutional Review Board. The approved IRB protocol number is 20-0070. All human serum specimens were obtained from the vaccinated subjects at the UTMB. All specimens were de-identified from patient information.

**Fluorescent foci reduction neutralization assay**. Neutralization titers of human and hamster sera were measured by fluorescent foci reduction neutralization assay (FFRNT) using the mNG SARS-CoV-2. Briefly, Vero E6 cells (2.5 × 10^4^) were seeded in each well of black μCLEAR flat-bottom 96-well plate (Greiner Bio-one™). The cells were incubated overnight at 37°C with 5% CO_2_. On the following day, each serum was 2-fold serially diluted in the culture medium with the first dilution of 1:10. The diluted serum was incubated with 100 PFU of mNG SARS-CoV-2 at 37 °C for 1 h (final dilution range of 1:10 to 1:20480), after which the serum-virus mixtures were inoculated onto Vero E6 cell monolayer in 96-well plates. After 1 h of infection, the inoculum was removed and 100 μl of overlay medium (DMEM supplemented with 0.8% methylcellulose, 2% FBS, and 1% P/S) was added to each well. The plates were incubated at 37°C for 20 h. The raw images were acquired using Cytation^TM^ 7 (BioTek) armed with 2.5× objective and processed using the default software setting. The foci in each well were counted and normalized to the non-serum-treated controls to calculate the relative infectivities. The curves of the relative infectivity versus the serum dilutions (log10 values) were plotted using Prism 9 (GraphPad). A nonlinear regression method was used to determine the dilution fold that neutralized 50% of mNG SARS-CoV-2 (defined as FFRNT). Each serum was tested in duplicates.

**Plaque reduction neutralization test (PRNT)**. A conventional 50% plaque-reduction neutralization test (PRNT_50_) was performed to measure the serum-mediated virus suppression as reported previously^5^. Individual sera were 2-fold serially diluted in culture medium with a starting dilution of 1:40 (dilution range of 1:40 to 1:1280). The diluted sera were incubated with 100 PFU of USA-WA1/2020 (WT) or mutant SARS-CoV-2. After 1 h incubation at 37°C, the serum-virus mixtures were inoculated onto 6-well plates with a monolayer of Vero E6 cells pre-seeded on the previous day. The minimal serum dilution that suppressed >50% of viral plaques is defined as PRNT_50_.

**Antigenic cartography**. A target distance from an individual serum to each virus was derived by calculating the difference between the logarithm (log2) reciprocal neutralization titer for that particular virus and the log2 reciprocal maximum titer achieved by that serum (against any virus). Thus, the higher the reciprocal titer, the shorter the target distance. As the log2 of the reciprocal titer was used, a 2-fold change in titer equates to a fixed change in target distance whatever the magnitude of the actual titers. Antigenic cartography^6^ then was used to optimize the positions of the viruses and sera relative to each other on a map, minimizing the sum-squared error between map distance and target distance. Each virus is therefore positioned by multiple sera, and the sera themselves also are positioned only by their distances to the viruses. Hence, sera with different neutralization profiles to the virus panel are in separate locations on the map but contribute equally to the positioning of the viruses.

**References**

1. Xie, X.*, et al.* Engineering SARS-CoV-2 using a reverse genetic system. *Nature Protocols* **16**, 1761-1784 (2021).

2. Xie, X.*, et al.* An Infectious cDNA Clone of SARS-CoV-2. *Cell Host Microbe* **27**, 841-848 e843 (2020).

3. Kurhade, C.*, et al.* Neutralization of Omicron BA.1, BA.2, and BA.3 SARS-CoV-2 by 3 doses of BNT162b2 vaccine. *BioRxiv*, <https://biorxiv.org/cgi/content/short/2022.2003.2024.485633v485631> (2022).

4. Xia, H.*, et al.* Neutralization and durability of 2 or 3 doses of the BNT162b2 vaccine against Omicron SARS-CoV-2. *Cell Host Microbe* **30**, 485-488.e483 (2022).

5. Muruato, A.E.*, et al.* A high-throughput neutralizing antibody assay for COVID-19 diagnosis and vaccine evaluation. *Nature Communications* **11**, 4059 (2020).

6. Smith, D.J.*, et al.* Mapping the antigenic and genetic evolution of influenza virus. *Science* **305**, 371-376 (2004).

**Supplementary figures**


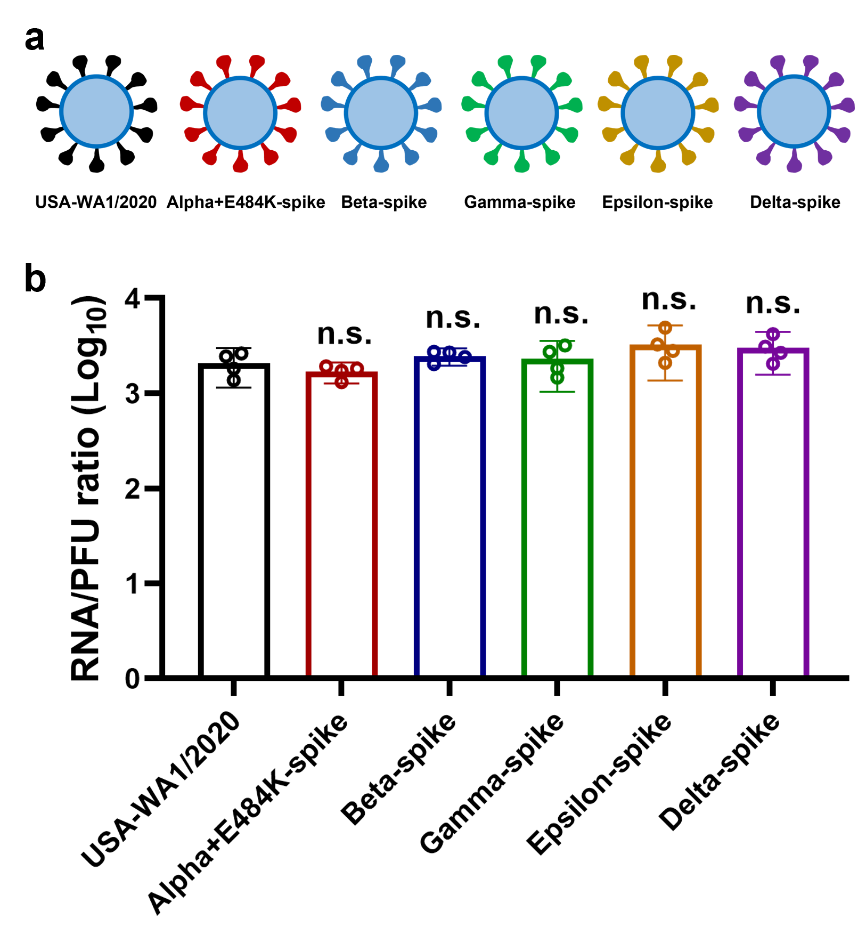


**Supplementary figure 1. The RNA/PFU ratios of different SARS-CoV-2 variants.**

(**a**) Chimeric USA-WA1/2020 bearing variant spikes. The spike genes from Alpha, Beta, Gamma, Epsilon, and Delta variants of SARS-CoV-2 were introduced into the USA-WA1/2020 backbone. (**b**) Ratios of viral genomic RNA versus plaque-forming unit (RNA/PFU) of SARS-CoV-2 spike variants. The genomic RNA and PFU of individual viral stocks were measured by RT-qPCR and plaque assay, respectively. The USA-WA1/2020 strain served as a control. Dots represent individual biological replicates from 4 aliquots of viruses. The means with 95% confidence intervals are shown. A non-parametric Mann-Whitney test was used to determine significant differences between USA-WA1/2020 and other variants. *P* values were adjusted using the Bonferroni correction to account for multiple comparisons. Differences were considered significant if *P* < 0.01; n.s., no statistical difference.


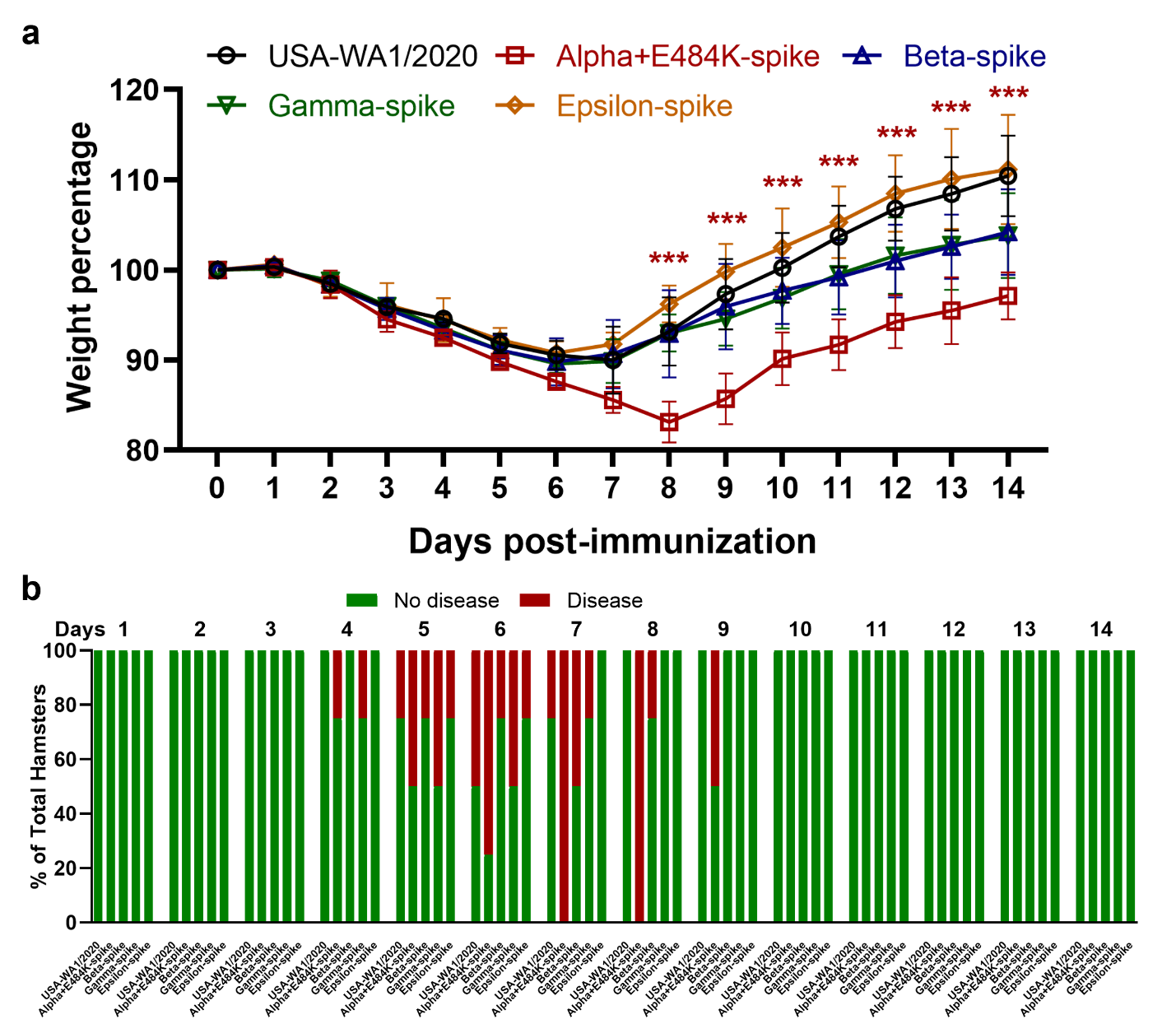


**Supplementary figure 2. Morbidity of hamsters after immunized with variant-spike SARS-CoV-2.**

(**a**) Hamster body weight loss after immunized with variant-spike SARS-CoV-2. The hamsters (n=4) were intranasally infected with 10^6^ PFU viruses. The body weights were measured daily from day 0 to day 14 days post-immunization. The weight loss data are shown as mean ± standard deviation and statistically analyzed using two-way ANOVA Turkey’s multiple comparisons. The red stars show the statistical significance (******* *P* < 0.001) between USA-WA1/2020-immunized hamsters and Alpha+E484K-spike-immunized hamsters. (**b**) Percentages of hamsters with or without diseases (including ruffled fur, lethargic, hunched, and reluctance to move when stimulated) from day 1 to day 14 post-immunization.


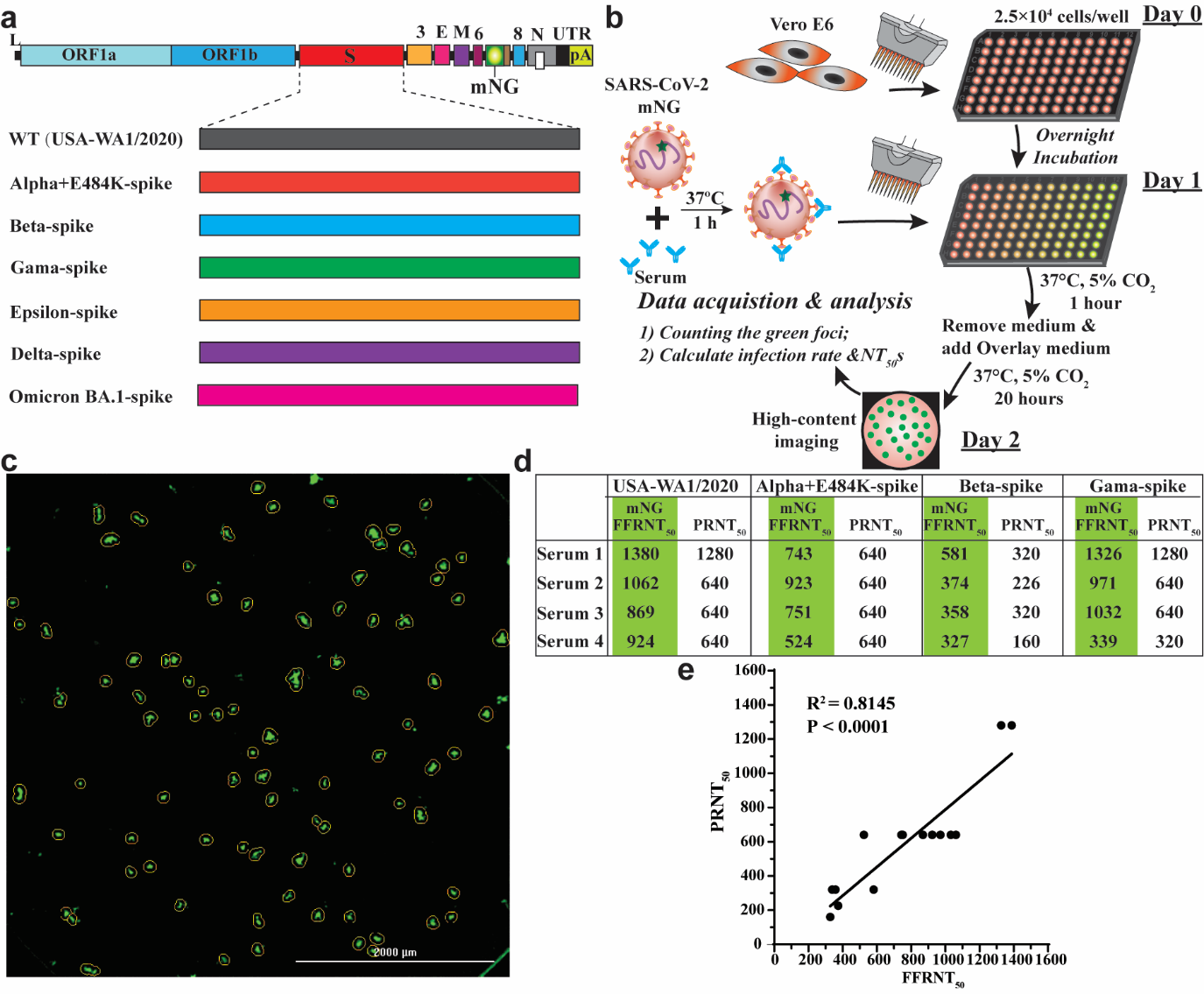


**Supplementary figure 3. Correlation between FFRNT_50_ and PRNT_50_.**

(**a**) Diagram of mNG USA-WA1/2020 and spike variants. mNG, mNeonGreen fluorescence protein gene. (**b**) Workflow of fluorescent foci reduction neutralization (FFRNT) assay. The details of the FFRNT assay were described in the Methods. (**c**) Representative images of foci formed in a 96-well plate after 20 h of infection. (**d**) FFRNT_50,_ and PRNT_50_ values for four human sera. The FFRNT_50_ values are shaded in green. (**e**) Correlation of FFRNT_50_ and PRNT_50_. The Pearson’s correlation coefficients and *P* values (two-tailed) are indicated.


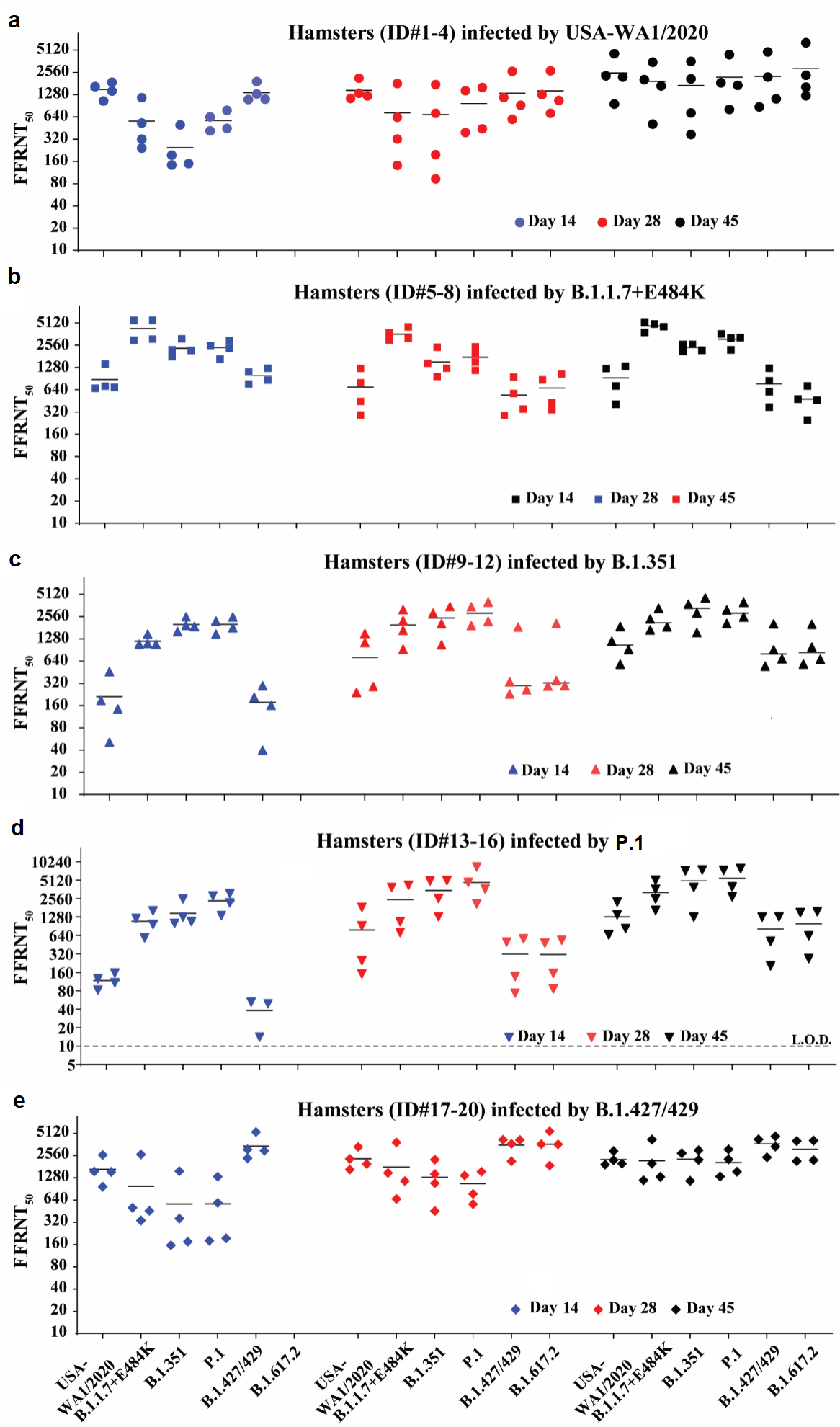


**Supplementary figure 4. FFRNT_50_s of hamster sera against mNG SARS-CoV-2 spike variants on days 14, 28, and 45 post-immunization.**

(**a-e**) Hamster (n=4 per group) were immunized with WT USA-WA1/2020 (**a**), Alpha+E484K-spike virus (**b**), Beta-spike virus (**c**), Gamma-spike virus (**d**), Epsilon-spike virus (**e**). Sera were collected on days 14, 28, and 45 post-immunization and tested for neutralizing activities against the indicated mNG viruses by FFRNT. The original FFRNT_50_ values are presented in **Supplementary tables 1-3.**


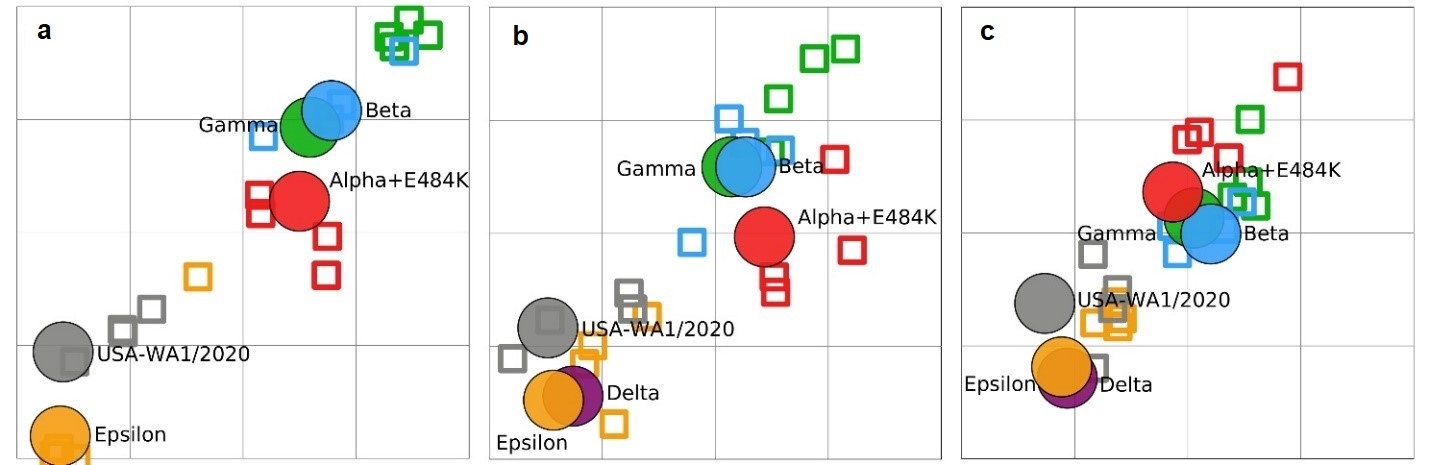


**Supplementary figure 5. Antigenic map of hamster serum titers against mNG reporter SARS-CoV-2 spike variants on days 14, 28, and 45 post-immunization.**

(**a-c**) Antigenic maps made from the FFRNT_50_ values presented at Day 14 (**a**), Day 28 (**b**), and Day 45 (**c**) in **Supplementary tables 1-3.** Antigens are shown as circles, sera by open squares. The vertical and horizontal axes both represent antigenic distance, and, because only the relative positions of antigens and sera can be determined, the orientation of the map within these axes is free. The spacing between grid lines corresponds to a two-fold dilution of antiserum in the assay. Two grids correspond to four-fold dilution, three to eight-fold dilution, and so on.


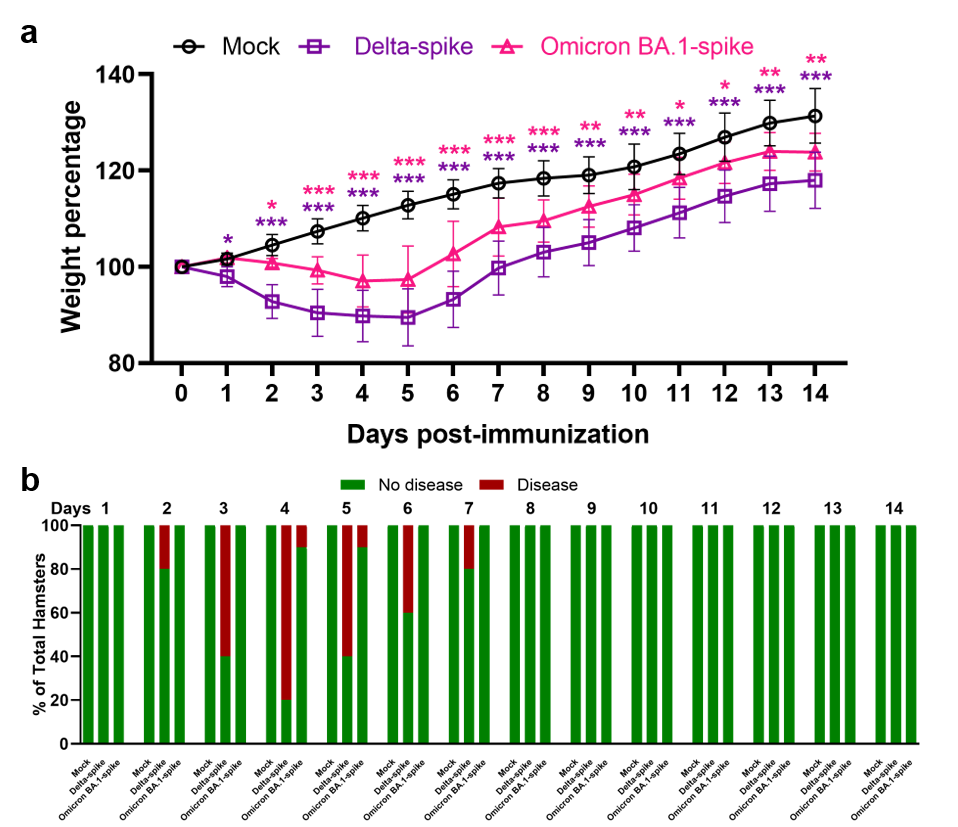


**Supplementary figure 6. Morbidity of hamsters after immunized with Delta- or Omicron BA.1-spike SARS-CoV-2.**

(**a**) Body weight loss after immunized with variant-spike SARS-CoV-2 viruses. The hamsters (n=10 for mock, n=9 for Delta-spike virus, n=10 for Omicron BA.1-spike virus) were intranasally infected with 10^6^ PFU viruses. The body weights were measured daily from days 0 to 14 post-immunization. The weight loss data are shown as mean ± standard deviation and statistically analyzed using two-way ANOVA Turkey’s multiple comparisons. The purple and pink stars show the statistical significance (* *P* < 0.05, ** *P* < 0.01, ******* *P* < 0.001) between Delta-spike or Omicron BA.1-spike virus-immunized hamsters and mock animals, respectively. (**b**) Percentages of hamsters with or without diseases (including ruffled fur, lethargic, hunched, and reluctance to move when stimulated) from days 1 to 14 post-immunization.

**
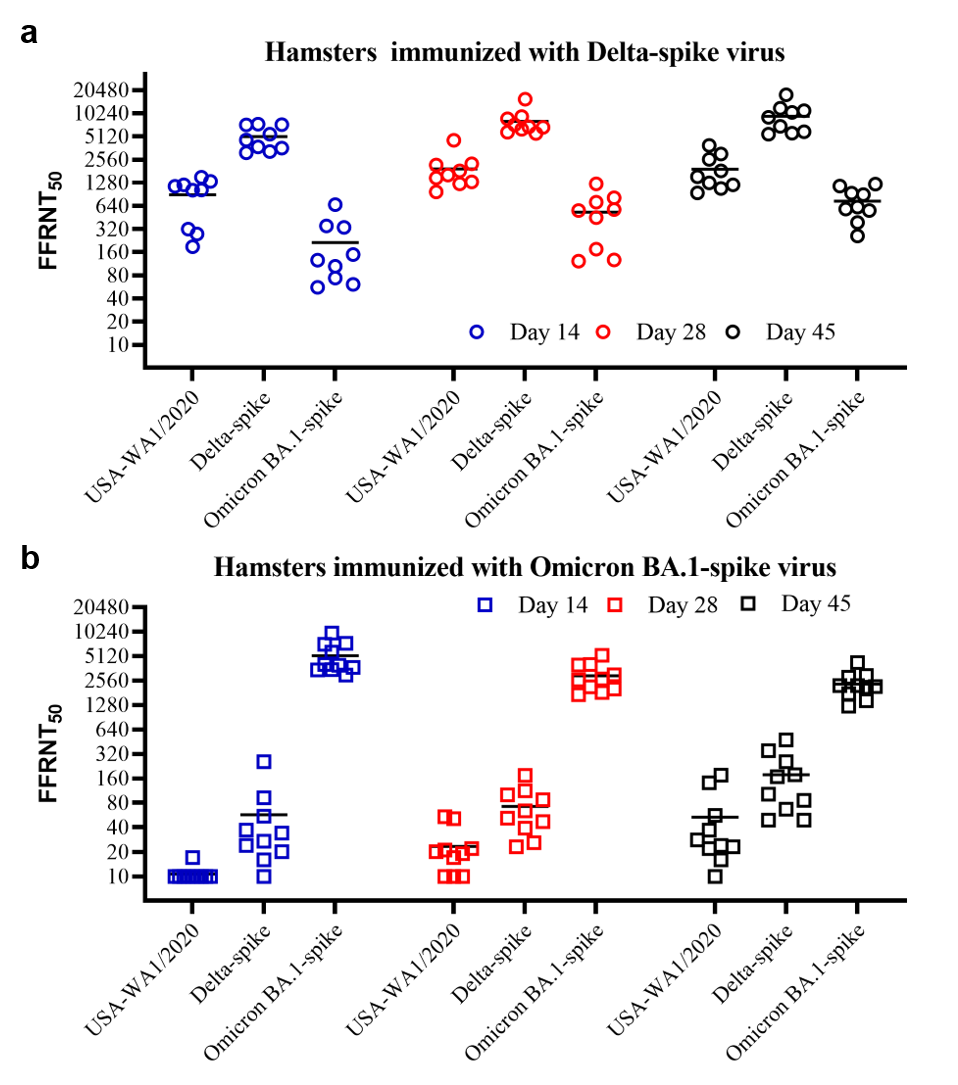
**

**Supplementary figure 7. FFRNT_50_s of hamster sera against mNG Delta- and Omicron BA.1-spike SARS-CoV-2 variants on days 14, 28, and 45 post-immunization.**

(**a,b**) Hamsters (n=10 for mock, n=9 for Delta-spike virus, n=10 for Omicron BA.1-spike virus) were immunized with Delta- (**a**) and Omicron BA.1-spike (**b**) viruses, respectively. Sera were collected on days 14, 28, and 45 post-immunization and tested for neutralization titers against the indicated mNG viruses by FFRNT. The original FFRNT_50_ values are presented in **Supplementary table 4.**

**Supplementary table 1. FFRNT_50_s of twenty hamster sera on day 14 post-immunization.**

| Virus for infection | Hamster ID | FFRNT_50_s against SARS-CoV-2 spike variants | | | | |
| --- | --- | --- | --- | --- | --- | --- |
|  |  | USA-WA1/2020 mNG | Alpha+E484K-spike mNG | Beta-spike mNG | Gamma-spike mNG | Epsilon-spike mNG |
| USA-WA1/2020 | 1 | 1048 | 320 | 194 | 446 | 1113 |
|  | 2 | 1875 | 1158 | 498 | 785 | 1916 |
|  | 3 | 1643 | 242 | 143 | 413 | 1304 |
|  | 4 | 1426 | 528 | 149 | 637 | 1099 |
|  | Mean | 1498 | 562 | 246 | 570 | 1358 |
| Alpha+E484K-spike | 5 | 689 | 5561 | 1789 | 1660 | 1111 |
|  | 6 | 669 | 5526 | 2214 | 2543 | 864 |
|  | 7 | 1431 | 2981 | 3122 | 2967 | 762 |
|  | 8 | 714 | 3087 | 2175 | 2318 | 1246 |
|  | Mean | 876 | 4289 | 2325 | 2372 | 996 |
| Beta-spike | 9 | 461 | 1079 | 1610 | 2226 | 296 |
|  | 10 | 188 | 1482 | 2537 | 2520 | 161 |
|  | 11 | 51 | 1082 | 1947 | 1486 | 40 |
|  | 12 | 144 | 1113 | 1881 | 1802 | 208 |
|  | Mean | 211 | 1189 | 1994 | 2009 | 176 |
| Gamma-spike | 13 | 108 | 1215 | 2525 | 2833 | 49 |
|  | 14 | 125 | 583 | 1006 | 1346 | <10 |
|  | 15 | 155 | 967 | 1075 | 2166 | 14 |
|  | 16 | 82 | 1617 | 1275 | 3092 | 52 |
|  | Mean | 118 | 1096 | 1470 | 2359 | 38 |
| Epsilon-spike | 17 | 966 | 337 | 156 | 194 | 2346 |
|  | 18 | 2602 | 502 | 358 | 583 | 5297 |
|  | 19 | 1535 | 457 | 175 | 179 | 2977 |
|  | 20 | 1552 | 2637 | 1572 | 1321 | 3085 |
|  | Mean | 1664 | 983 | 565 | 569 | 3426 |

**Supplementary table 2. FFRNT_50_s of twenty hamster sera on day 28 post-immunization.**

| Virus for infection | Hamster ID | FFRNT_50_s against SARS-CoV-2 spike variants | | | | | |
| --- | --- | --- | --- | --- | --- | --- | --- |
|  |  | USA-WA1/2020 mNG | Alpha+E484K-spike mNG | Beta-spike mNG | Gamma-spike mNG | Epsilon-spike mNG | Delta-spike mNG |
| USA-WA1/2020 | 1 | 1226 | 632 | 714 | 1601 | 1167 | 1278 |
|  | 2 | 2140 | 1807 | 1749 | 1442 | 2646 | 2691 |
|  | 3 | 1137 | 141 | 93 | 392 | 920 | 713 |
|  | 4 | 1336 | 321 | 197 | 442 | 594 | 1068 |
|  | Mean | 1460 | 725 | 688 | 969 | 1332 | 1438 |
| Alpha+E484K-spike | 5 | 445 | 3007 | 963 | 1169 | 288 | 430 |
|  | 6 | 1239 | 4511 | 1453 | 1920 | 950 | 1044 |
|  | 7 | 792 | 3174 | 1248 | 1507 | 569 | 867 |
|  | 8 | 291 | 3787 | 2405 | 2425 | 349 | 343 |
|  | Mean | 692 | 3620 | 1517 | 1755 | 539 | 671 |
| Beta-spike | 9 | 1511 | 3184 | 3494 | 4027 | 1850 | 2068 |
|  | 10 | 241 | 1671 | 2048 | 2209 | 230 | 293 |
|  | 11 | 1144 | 2253 | 2848 | 3473 | 336 | 349 |
|  | 12 | 290 | 931 | 1059 | 1932 | 261 | 299 |
|  | Mean | 797 | 2010 | 2362 | 2910 | 669 | 752 |
| Gamma-spike | 13 | 914 | 3874 | 5009 | 4685 | 564 | 480 |
|  | 14 | 1837 | 4197 | 4954 | 8391 | 498 | 535 |
|  | 15 | 247 | 707 | 1282 | 2090 | 73 | 85 |
|  | 16 | 150 | 1068 | 2555 | 3644 | 137 | 153 |
|  | Mean | 787 | 2462 | 3450 | 4703 | 318 | 313 |
| Epsilon-spike | 17 | 1961 | 1484 | 1080 | 770 | 3683 | 5442 |
|  | 18 | 3347 | 1153 | 1432 | 1373 | 4163 | 3634 |
|  | 19 | 1654 | 662 | 455 | 562 | 2132 | 1868 |
|  | 20 | 2298 | 3833 | 2251 | 1545 | 4182 | 3605 |
|  | Mean | 2315 | 1783 | 1305 | 1063 | 3540 | 3637 |

**Supplementary table 3. FFRNT_50_s of twenty hamster sera on day 45 post-immunization.**

| Virus for infection | Hamster ID | FFRNT_50_s against SARS-CoV-2 spike variants | | | | | |
| --- | --- | --- | --- | --- | --- | --- | --- |
|  |  | USA-WA1/2020 mNG | Alpha+E484K-spike mNG | Beta-spike mNG | Gamma-spike mNG | Epsilon-spike mNG | Delta-spike mNG |
| USA-WA1/2020 | 1 | 4574 | 3509 | 3590 | 4437 | 4843 | 6410 |
|  | 2 | 2299 | 2042 | 2094 | 1850 | 2207 | 2337 |
|  | 3 | 953 | 510 | 370 | 807 | 1125 | 1612 |
|  | 4 | 2205 | 1682 | 723 | 1707 | 871 | 1232 |
|  | Mean | 2508 | 1936 | 1694 | 2200 | 2262 | 2898 |
| Alpha+E484K-spike | 5 | 718 | 3794 | 2628 | 3627 | 601 | 462 |
|  | 6 | 1326 | 5262 | 2624 | 3211 | 1248 | 719 |
|  | 7 | 1237 | 4554 | 2115 | 3225 | 844 | 477 |
|  | 8 | 405 | 4930 | 2181 | 2210 | 372 | 248 |
|  | Mean | 922 | 4635 | 2387 | 3068 | 766 | 477 |
| Beta-spike | 9 | 1881 | 3305 | 4574 | 3994 | 2047 | 2005 |
|  | 10 | 582 | 1852 | 2857 | 2527 | 545 | 584 |
|  | 11 | 1184 | 2374 | 3746 | 3139 | 906 | 988 |
|  | 12 | 920 | 1692 | 1554 | 2068 | 688 | 680 |
|  | Mean | 1142 | 2306 | 3183 | 2932 | 1047 | 1064 |
| Gamma-spike | 13 | 1391 | 3638 | 7481 | 7345 | 1272 | 1505 |
|  | 14 | 2251 | 5095 | 7255 | 7892 | 1282 | 1566 |
|  | 15 | 656 | 1638 | 1274 | 2734 | 203 | 268 |
|  | 16 | 828 | 2521 | 3882 | 3986 | 514 | 633 |
|  | Mean | 1282 | 3223 | 4973 | 5489 | 818 | 993 |
| Epsilon-spike | 17 | 1982 | 1315 | 2236 | 1548 | 3379 | 2149 |
|  | 18 | 2205 | 1991 | 2742 | 3084 | 4212 | 4069 |
|  | 19 | 1928 | 1182 | 1158 | 1326 | 2419 | 2208 |
|  | 20 | 2930 | 4189 | 3004 | 2268 | 4641 | 4016 |
|  | Mean | 2261 | 2169 | 2285 | 2057 | 3663 | 3111 |

**Supplementary table 4. FFRNT_50_s of Delta- and Omicron BA.1-spike immunized hamster sera post-immunization.**

| Virus for infection | Hamster ID | FFRNT_50_s against SARS-CoV-2 spike variants | | | | | | | | |
| --- | --- | --- | --- | --- | --- | --- | --- | --- | --- | --- |
|  |  | Day 14 | | | Day 28 | | | Day 45 | | |
|  |  | USA-WA1/2020 mNG | Delta-spike mNG | Omicron BA.1-spike mNG | USA-WA1/2020 mNG | Delta-spike mNG | Omicron BA.1-spike mNG | USA-WA1/2020 mNG | Delta-spike mNG | Omicron BA.1-spike mNG |
| Delta-spike | 21 | 1025 | 7374 | 337 | 1314 | 6787 | 716 | 1817 | 5871 | 389 |
|  | 22 | 276 | 3732 | 105 | 1479 | 6703 | 556 | 1273 | 10509 | 259 |
|  | 23 | 1198 | 4597 | 350 | 969 | 8686 | 449 | 944 | 9080 | 1160 |
|  | 24 | 188 | 3139 | 126 | 1234 | 9241 | 574 | 1205 | 6928 | 558 |
|  | 25 | 1033 | 3602 | 61 | 1614 | 5814 | 122 | 3012 | 11873 | 583 |
|  | 26 | 318 | 3243 | 56 | 2179 | 5593 | 127 | 2566 | 11060 | 616 |
|  | 27 | 1326 | 7204 | 74 | 2257 | 7208 | 175 | 1528 | 5461 | 935 |
|  | 28 | 1504 | 7259 | 149 | 1820 | 6347 | 812 | 3908 | 17817 | 1227 |
|  | 29 | 1153 | 5472 | 665 | 4555 | 15583 | 1240 | 1077 | 5643 | 897 |
|  | Mean | 891 | 5069 | 214 | 1936 | 7996 | 530 | 1926 | 9360 | 736 |
| Omicron BA.1-spike | 30 | 17 | 24 | 3477 | 51 | 174 | 2140 | 176 | 352 | 2098 |
|  | 31 | 10 | 34 | 7166 | 10 | 113 | 4020 | 23 | 178 | 2942 |
|  | 32 | 10 | 20 | 3701 | 54 | 100 | 3006 | 142 | 476 | 2815 |
|  | 33 | 10 | 55 | 3940 | 17 | 26 | 1716 | 10 | 49 | 1448 |
|  | 34 | 10 | 27 | 9878 | 20 | 87 | 5260 | 28 | 258 | 4258 |
|  | 35 | 10 | 10 | 5758 | 10 | 52 | 3983 | 56 | 168 | 2155 |
|  | 36 | 10 | 257 | 4019 | 10 | 23 | 2597 | 37 | 67 | 2219 |
|  | 37 | 10 | 37 | 3002 | 21 | 64 | 1832 | 16 | 102 | 1241 |
|  | 38 | 10 | 16 | 7380 | 19 | 39 | 2687 | 22 | 86 | 2215 |
|  | 39 | 10 | 92 | 3468 | 22 | 47 | 2022 | 24 | 49 | 1764 |
|  | Mean | 11 | 57 | 5179 | 23 | 73 | 2926 | 53 | 179 | 2316 |
